# Do Seasons Have an Influence on the Incidence of Depression? The Use of an Internet Search Engine Query Data as a Proxy of Human Affect

**DOI:** 10.1371/journal.pone.0013728

**Published:** 2010-10-28

**Authors:** Albert C. Yang, Norden E. Huang, Chung-Kang Peng, Shih-Jen Tsai

**Affiliations:** 1 Department of Psychiatry, Chu-Tung Veterans Hospital, Jhudong Township, Taiwan; 2 Divisions of Psychiatry, School of Medicine, National Yang-Ming University, Taipei, Taiwan; 3 Institute of Clinical Medicine, National Yang-Ming University, Taipei, Taiwan; 4 Research Center for Adaptive Data Analysis, National Central University, Chungli, Taiwan; 5 Division of Interdisciplinary Medicine and Biotechnology and Margret and H. A. Rey Institute for Nonlinear Dynamics in Medicine, Beth Israel Deaconess Medical Centre/Harvard Medical School, Boston, Massachusetts, United States of America; 6 Wyss Institute for Biologically Inspired Engineering at Harvard University, Boston, Massachusetts, United States of America; 7 Department of Psychiatry, Taipei Veterans General Hospital, Taipei, Taiwan; University of Southampton, United Kingdom

## Abstract

**Background:**

Seasonal depression has generated considerable clinical interest in recent years. Despite a common belief that people in higher latitudes are more vulnerable to low mood during the winter, it has never been demonstrated that human's moods are subject to seasonal change on a global scale. The aim of this study was to investigate large-scale seasonal patterns of depression using Internet search query data as a signature and proxy of human affect.

**Methodology/Principal Findings:**

Our study was based on a publicly available search engine database, Google Insights for Search, which provides time series data of weekly search trends from January 1, 2004 to June 30, 2009. We applied an empirical mode decomposition method to isolate seasonal components of health-related search trends of depression in 54 geographic areas worldwide. We identified a seasonal trend of depression that was opposite between the northern and southern hemispheres; this trend was significantly correlated with seasonal oscillations of temperature (USA: r = −0.872, p<0.001; Australia: r = −0.656, p<0.001). Based on analyses of search trends over 54 geological locations worldwide, we found that the degree of correlation between searching for depression and temperature was latitude-dependent (northern hemisphere: r = −0.686; p<0.001; southern hemisphere: r = 0.871; p<0.0001).

**Conclusions/Significance:**

Our findings indicate that Internet searches for depression from people in higher latitudes are more vulnerable to seasonal change, whereas this phenomenon is obscured in tropical areas. This phenomenon exists universally across countries, regardless of language. This study provides novel, Internet-based evidence for the epidemiology of seasonal depression.

## Introduction

Seasonality, which is driven by variation in solar influx, is one of the most dramatic environmental variables that affects the physical and biological properties of life. The recognition that humans are subject to seasonal changes in mood and behavior may date back to ancient times, when Hippocrates observed variations in seasonal incidences of melancholy and mania [1]. Poets have often portrayed a sense of sadness that may accompany the shortening days of fall and winter. Biologically, seasonal rhythms have been identified in many human social behaviors and in functions including weight, appetite, sleep, birth, death, and mood [2], [3], [4], [5], [6], [7], [8]. Studies of recurrent seasonal depression also have enhanced interest in the clinical relevance of such seasonal changes [9], [10], [11], [12], [13], [14]. Although it is commonly believed that human beings are affected by seasonality, no study has investigated the seasonal patterns of depression on a global scale.

The Internet has become an important information source in recent years. Keyword-driven Internet search engines allow billions of people worldwide to have easy, instant access to a vast and diverse amount of information online. These search records, when properly archived and de-identified, are the largest dataset ever seen in human history and are priceless to scientific researchers in many fields [15], [16]. For example, Internet search query data have been demonstrated to predict influenza epidemics [17], [18], [19], other infectious diseases [20], or unemployment rate [21]. Only recently, these search query data were available to the public using programs such as Google Insights for Search [22], a free service provided by Google Incorporation that allows researchers to examine trends of certain search keywords. This web-based service provides de-identified, normalized weekly trend data of certain keyword's search volumes and opens a unique window to investigate collective human behaviors on the Internet on a gigantic scale.

Here, we present a study based on search trend data from Google Insights for Search. Search interests of certain keywords such as “shirt” and “sweater” are obviously seasonally-dependent. Search queries for other words, such as those that convey emotional sense or medical meanings, may also reflect seasonal patterns of human behavior or illness. Because fluctuation in search trend data usually consists of multiple periodic components with characteristics of non-stationary and non-linearity, an adaptive-based method, such as empirical mode decomposition (EMD) analysis, can be useful in isolating meaningful seasonal components [23], [24]. The EMD method provides a generic algorithm to decompose a complex time series into a set of intrinsic oscillations, called intrinsic mode functions (IMFs), which oscillate at different time scales and are orthogonal to each other. We applied the EMD method to isolate a seasonal IMF in Internet search trends derived from 54 geographic locations worldwide. We aimed to test the following two hypotheses. (1) The Internet searches for depression fluctuate seasonally, with increased search activity during the respective winter season in the northern or southern hemisphere. (2) The seasonality of Internet searches for depression will depend in part on relative latitude; that is, those searches that originate from high latitude areas (i.e., remote from the equator) will be more seasonal.

## Results

### Decomposition of search trend data

**Figure 1:**
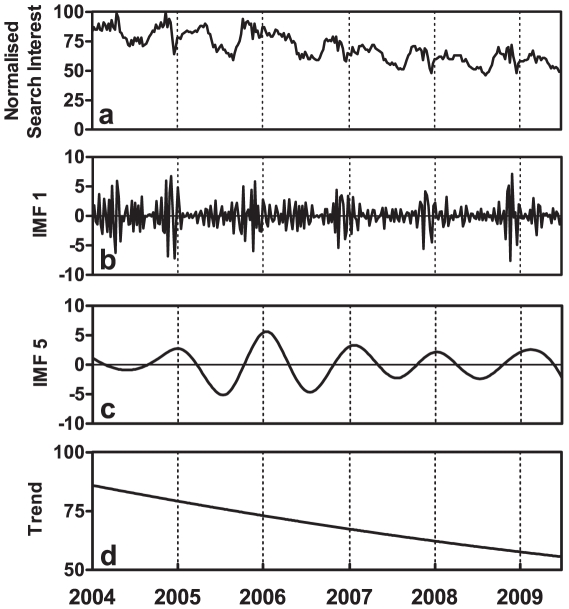
Examples of decomposition using the EMD method: A) Time series of weekly normalized search interests of health-related queries for depression in the United States from 2004–2009 (287 data points). B) Time series of first IMF, which is a component of noise by statistical test. C) Time series of seasonal IMF. D) Residual component (overall trend). The seasonal IMF is the most energetic components in these data.

Figure 1 shows the decomposition of search trends for the keyword *depression* in the northern hemisphere; these data are limited to those search queries under the health category that originated within the United States of America (USA; see Figure S1 for full set of IMFs). The highest energetic IMFs embedded in this search trend data are the IMF associated with seasonal oscillations (Figure 1c; and power spectrum in Figure S2). The combination of this seasonal IMF and the residual component (overall trend) accounts for 73.0% (95% confidence interval (C.I.) 60.6–85.3%) of the variability in raw search trend data. The seasonal IMF was not a component of noise (p<0.01) and therefore may contain information that can elucidate their physical meanings.

### Search trend of depression in northern and southern hemispheres

**Figure 2:**
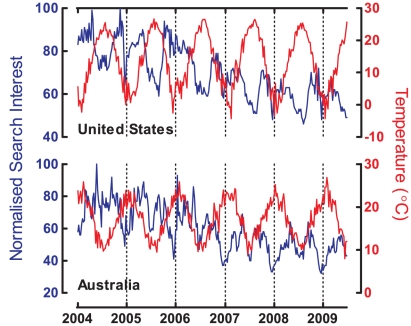
Comparison of raw data between search trend of depression (blue line) and temperature (red line) in the northern hemisphere (United States of America) and the southern hemisphere (Australia).

**Figure 3:**
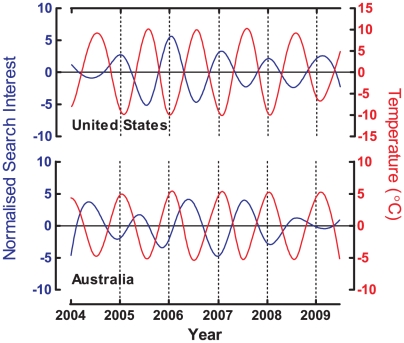
Comparison of decomposed seasonal IMF between search trend of health-related queries for depression (blue line) and temperature (red line) in the northern hemisphere (United States of America) and the southern hemisphere (Australia).

Because the seasons in the southern hemisphere are opposite to those in the northern hemisphere, we examined seasonal patterns by comparing search trends of depression in the northern and southern hemispheres. We based our information on nationwide search data from the USA and Australia, countries used to represent the northern and southern hemispheres, respectively. Using temperature data as a seasonal reference, Figure 2 demonstrates that the raw data of depression trends in the USA and Australia were opposite each other, and both peaked in the respective winter season. By applying the EMD method to isolate the seasonal IMF in search trends and temperature data, Figure 3 shows that the seasonal IMFs of search trends in both countries are negatively correlated with seasonal fluctuations in temperature (USA: r = −0.872, p<0.001; Australia: r = −0.656, p<0.001). It is interesting to note that we also found seasonal patterns in other medical or psychiatric terms (e.g., angina, common cold, or allergy; data not shown).

### Comparison of search trend of depression at global-wide scale

**Figure 4:**
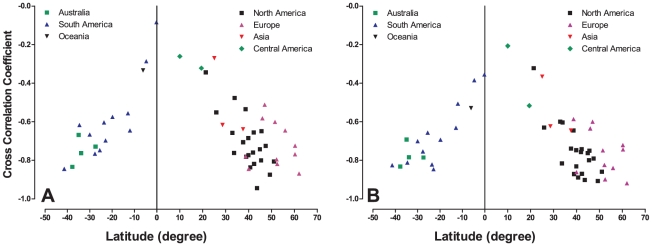
The relationship between seasonality of Internet search trend of depression and geographic locations. Comparisons are made between seasonal IMF of search trends, temperature and solar radiation. Each data point represents a geographic area and is categorized by its respective continent/land. For the coordinate of the data point, each geographic location's latitude is the x-coordinate, and the y-coordinate is the cross-correlation coefficient between its seasonal IMF of local search trend and that of (a) temperature as well as (b) solar radiation.

To investigate the search trend of depression on a global scale, we gathered local search trend data of health-related queries for depression originating from 54 geographic locations in the northern and southern hemispheres (Table S1). Equivalent words representing depression were used in searches of non-English speaking countries (Table S2). By estimating a cross-correlation coefficient between seasonal IMFs of search trend and temperature (see Table S3 for raw correlation values), Figure 4a shows a linear relationship between latitude and the correlation between temperature and search interests for depression (northern hemisphere: r = −0.686; p<0.001; southern hemisphere: r = 0.871; p<0.0001).

Because temperature change is mainly driven by exposure to sunlight and evidence has been found supporting the sunlight-mediated change in the prevalence of depression, a solar influx model was used to approximate the daylight received at a given location and time in a year [25]. By estimating a cross-correlation coefficient between seasonal IMFs of search trend and solar influx data (see Table S3 for raw correlation values), Figure 4b also shows a linear relationship between latitude and the correlation between solar influx data and search interests for depression (northern hemisphere: r = −0.738; p<0.001; southern hemisphere: r = 0.864; p<0.001).

## Discussion

The key finding emerging from this study is that health-related Internet search queries for depression are significantly correlated with temperature; this finding was evidenced by the fact that increased search activity was found during respective winter season in the northern and southern hemisphere. We demonstrate that this correlation (i.e., seasonality) is latitude-dependent, based on analysis of search trends over 54 geological locations worldwide. This phenomenon also exists universally across countries, regardless of language. Using Internet search query data as a signature of search interests in depression, our findings support the common notion that people in higher latitudes are more vulnerable to low mood during the winter season than those in tropical areas.

There are several implications of our findings. First, the Internet has become an important source of medical information in recent years [26] and is increasingly influencing not only the practice of clinical medicine but also the population in general [27]. Our findings of seasonal patterns are not exclusive to depression. Investigation of seasonal patterns in Internet search trends may aid future research in many disciplines, including epidemiology, sociology, or health care economy. For example, a specialized search query database has been used to predict influenza epidemics [17]. With the appropriate regulation and protection of privacy of Internet search records, an effective, reliable prediction system for many important medical illnesses or psychiatric emergencies (e.g., suicide) can be established. Second, prior studies regarding prevalence of seasonal mood disorders in different latitude have yielded inconsistent results [28], [29], [30]. Using Internet data as a proxy of human affect, our analyses complement traditional approaches to epidemiological research on seasonal depression. The seasonal IMF significantly accounted for variability in raw search trend data (e.g., 73% of searches in USA); its connection with seasonal depressive disorder may warrant future research.

Traditional methods of time series decomposition include cosinor analysis, seasonal decomposition of time series by local regression, or autoregressive algorithms. These methods require either predefined frequency of oscillations or the assumption of stationarity, which are often invalid in epidemiologic time series. EMD is empirical and adaptive and does not require any predetermined assumptions of data. Thus, it is useful in isolating physically meaningful oscillations embedded in complex raw data [24]. For example, EMD has been applied to isolate travelling waves in dengue hemorrhagic fever incidences across Thailand [31] and to evaluate the risk of stroke by identifying oscillations in cerebral blood flow related to cerebral auto-regulations [32]. We also have applied this method to delineate the association of suicide with air pollution and meteorological variables [33]. We propose that the analysis and scope presented in this study provides a more generalized method to analyze health-related issues using an Internet search query database.

The biological mechanisms underlying the seasonality of Internet search for depression are not understood at present. Several classes of mechanisms have been proposed in studying the neurobiology of depression as it relates to seasonal change. Indolamines, including tryptophan, serotonin and melatonin, have important roles of transducing light signals from the environment into cells [34] and in signaling seasonal changes in humans [35]. Functional imaging studies have found higher serotonin transporter binding during winter, which may facilitate extracellular serotonin loss and eventually lead to lower mood [36]. Our findings that search interests of depression were higher during colder periods, with respect to corresponding time in northern and southern hemispheres, are consistent with this biological evidence.

The interpretations made in this study have limitations. Individual search queries for depression (or other medical terms) cannot accurately reflect the actual mood state (or severity of medical conditions) of Internet users. Factors other than seasonal changes, including news events, cultural differences or alcohol consumption, might influence human affect and thus Internet search behaviors. However, consistent with a prior study of detecting influenza epidemics using Internet search data [17], it is rational to assume that the reason people seek health information about depression on the Internet is because they or people they know may be experiencing mood disturbances. The collective phenomenon of Internet search behavior is unlikely to be consciously manipulated by a single user and can be a meaningful, robust symbol of human behaviors or disease patterns across large populations.

In conclusion, our analysis provides novel, Internet-based evidence regarding the epidemiology of seasonal depression. The Internet only began about two decades ago, and public search trend databases have only recently become available; therefore, extensive analysis of Internet search data emerging over a longer time scale in relation to health, social, economic, and environmental factors is an important area for future research.

## Materials and Methods

### Search trend data

De-identified, normalized weekly search trend data (see supplementary Dataset S1) were retrieved publicly via an online service called Google Insights for Search. The use of data and relevant privacy rules were adhered to the service terms of Google Inc. (www.google.com/insights/terms.html). None of the data in this study contain personal information or individualized records of Internet search history. We considered only search trend data that were non-zero and continuously available from January 1, 2004 to June 30, 2009. Using the keyword *depression*, local search trend data from 54 geographic locations were retrieved, including both English-speaking and non-English speaking countries (Table S1). Standard translations of the keyword *depression* were used to retrieve search trends from non-English speaking countries (Table S2). To prevent the mixing of search queries that originated from unrelated regions or that contained multiple meanings, options for both regions and health-specific categories were specified accordingly when the data were downloaded. The study was approved by the institutional review boards of the Taipei Veterans General Hospital (Taipei, Taiwan).

### Temperature and solar influx data

Surface temperature data were obtained from two public climate data archives: the National Oceanic and Atmospheric Administration [37] and the British Atmospheric Data Centre [38]. Temperature time series was averaged on a weekly basis. For the 54 geographic locations used in this study (Table S1), temperature was measured at the corresponding capital city of each location. It should be noted that temperature data averaged from four cities in Australia and from seventeen cities in the USA (Table S1) were used in Figures 2 and 3 to compare these data with nationwide search trend data.

We applied a solar influx model [25] based on the latitude and longitude of the capital city to approximate the amount of daylight received at the 54 geographic locations. Publicly available software (http://www.ecy.wa.gov/programs/eap/models/; by Pelletier G at Washington State Department of Ecology, Olympia, WA, USA) was used to generate weekly time series of solar radiation at given locations.

### Empirical mode decomposition

The EMD method was developed to de-trend and identify intrinsic oscillations embedded in a complex signal [23]; this method has been widely applied in multiple disciplines [31], [32], [33], [39]. Unlike Fourier-based time series analysis, EMD holds no *a priori* assumptions for underlying structures of the time series and is therefore suitable for analyzing time series that consist of multiple periodic components (i.e., climate data or biomedical signals). The decomposition is based on the simple assumption that all data consist of a finite number of intrinsic components of oscillations. Each component of oscillation, termed IMF, was sequentially decomposed from the original time series by a sifting process. Each IMF has a characteristic time scale, making it suitable for isolating the seasonal component in the search trend data.

Briefly, the sifting process involves the following steps: 1) connecting local maxima or minima of a targeted signal to form the upper and lower envelopes by natural cubic spline lines, respectively; 2) extracting the first prototype IMF by estimating the difference between the targeted signal and the mean of the upper and lower envelopes; and 3) repeating the above procedures to produce a set of IMFs represented by a certain frequency-amplitude modulation at a characteristic time scale. The decomposition process is completed when no more IMFs can be extracted, and the residual component is treated as the overall trend of the raw data. Although these IMFs are empirically determined, they remain orthogonal to one another and may therefore contain independent physical meaning that is relevant to other parameters [24], [40].

To overcome the problem of scale mixing, a new noise-assisted method was employed to improve EMD: the ensemble EMD [41], [42]. This method defines the true IMF component as the average of an ensemble of trials (N = 1000 in our study), each consisting of the signal plus a white noise of finite amplitude. The standard deviation (SD) of added white noise is 3/10 of the SD in the original time series. The added noise in each trial is cancelled out in the ensemble mean of large trials. Of note, the uniformly added white noise helps to project the decomposition of IMFs onto comparable scales independent of the nature of original signals, thus reducing the problem of scale mixing. Although the noise introduced by the ensemble EMD algorithm may induce distortions to IMFs, the degree of distortion can be reduced by a large number of trials and is estimated as 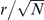, which is equal to 0.0095 in our study (where r is the amplitude ratio of noise to signal). A publicly available EMD algorithm was used in this study (http://rcada.ncu.edu.tw/research1.htm).

### Statistical analysis

Matlab software (version 2007; The Mathworks, Natick, Massachusetts) was used for numerical and statistical analyses. To validate and distinguish whether an IMF was a component of noise, a white noise null hypothesis [43] was used to assess statistical significance of IMFs that were decomposed from search trend data. If an IMF was rejected by the noise hypothesis, then it would contain non-noise fluctuations, which may have certain physical meanings (i.e., seasonal changes). After isolating and validating the seasonal IMF, multiple linear regression analysis was performed to estimate how much of the total variation in the search trend data could be explained by the combination of decomposed, seasonal IMFs.

To study the effect of latitude on magnitude of seasonality of search trends, we measured the correlation of amplitudes between search trend IMFs and temperature. Cross-correlation was employed to compute the best possible correlation between search trends and temperature/solar influx variables within limited time lags (eight weeks; equivalent to eight data points in this study). A *p* value of less than .05 (two-tailed) was required for statistical significance.

## Supporting Information

Table S1 List of 54 geographic areas with search trend data of depression.
(0.11 MB DOC)

Table S2 List of equivalent words representing the search term “depression” in non-English languages.
(0.05 MB DOC)

Table S3 Cross-correlation coefficient between seasonal IMF of local search trend and that of temperature as well as solar radiation.
(0.01 MB PDF)

Figure S1 Empirical mode decomposition of search interest time series for depression within the United States, Jan 1 2004 through Jun 30 2009.
(2.27 MB TIF)

Figure S2 Power spectrum of search interest time series for depression within the United States. The spectrum was derived from Hilbert transform. The most energetic intrinsic mode function (IMF) is the fifth IMF, which corresponds to seasonal oscillations of search interests over time. Of note, the fifth IMF shows a stable frequency modulation at around one cycle per year through the entire period.
(0.40 MB EPS)

Dataset S1 Raw dataset of search trends of depression.
(0.04 MB ZIP)
