# Supplementary material for: Do Seasons Have an Influence on the Incidence of Depression? The Use of an Internet Search Engine Query Data as a Proxy of Human Affect
Source: PLoS One. 2010 Oct 28;5(10):e13728. doi: 10.1371/journal.pone.0013728 (PMC2965678; doi:10.1371/journal.pone.0013728)
Supplement: Table S1 — List of 54 geographic areas with search trend data of depression. (0.11 MB DOC) [file pone.0013728.s001.doc]

**Table S1**. List of 54 geographic areas with search trend data of depression

| **Country** | **Representative Area/City** | **Language** | **Latitude** | **Longitude** |
| --- | --- | --- | --- | --- |
| Argentina | Buenos Aires | Spanish | 34 35 S | 058 29 W |
| Australia | New South Wales | English | 33 52 S | 151 12 E |
| Australia | Queensland | English | 27 23 S | 153 06 E |
| Australia | South Australia | English | 34 56 S | 138 31 E |
| Australia | Victoria | English | 37 49 S | 144 58 E |
| Brazil | Bahia | Portuguese | 12 54 S | 038 20 W |
| Brazil | Minas Gerais | Portuguese | 19 48 S | 042 09 W |
| Brazil | Parana | Portuguese | 25 31 S | 049 10 W |
| Brazil | Rio de Janeiro | Portuguese | 22 55 S | 043 10 W |
| Brazil | Rio Grande do Sul | Portuguese | 30 00 S | 051 11 W |
| Brazil | Santa Catarina | Portuguese | 27 40 S | 048 33 W |
| Brazil | Sao Paulo | Portuguese | 23 37 S | 046 39 W |
| Canada | Alberta | English | 51 06 N | 114 22 W |
| Canada | British Columbia | English | 49 18 N | 123 07 W |
| Canada | Ontario | English | 43 37 N | 079 21 W |
| Canada | Quebec | English | 45 28 N | 073 45 W |
| Chile | Puerto Montt | Spanish | 41 25 S | 073 05 W |
| Colombia | Bogotá | Spanish | 04 42 N | 074 08 W |
| Table S1. Continued | | | | |
| Costa Rica | San Jose | Spanish | 10 00 N | 084 13 W |
| Ecuador | Quito | Spanish | 00 09 S | 078 29 W |
| Finland | Helsinki | Finish | 60 15 N | 025 03 E |
| France | Entire region | French | 46 00 N | 002 00 E |
| Germany | Entire region | Deutsch | 52 28 N | 013 18 E |
| Indonesia | Jakarta | Indonesian | 06 06 S | 106 52 E |
| India | Entire region | English | 28 35 N | 077 12 E |
| South Korea | Seoul | Korean | 37 34 N | 126 58 E |
| Mexico | Mexico City | Spanish | 19 24 N | 099 12 W |
| Norway | Oslo | Norwegian | 60 12 N | 011 05 E |
| Portugal | Lisbon | Portuguese | 38 43 N | 009 09 W |
| Poland | Warsaw | Polish | 52 10 N | 020 58 E |
| Peru | Lima | Spanish | 12 00 S | 077 07 W |
| Switzerland | Entire region | Deutsch | 47 00 N | 008 00 E |
| Spain | Entire region | Spanish | 40 00 N | 004 00 W |
| Sweden | Stockholm | Swedish | 62 00 N | 015 00 E |
| Taiwan | Entire region | Chinese | 25 02 N | 121 32 E |
| United Kingdom | England | English | 51 30 N | 000 07 W |
| United Kingdom | Scotland | English | 55 57 N | 003 21 W |
| Table S1. Continued | | | | |
| United States | Atlanta | English | 33 39 N | 084 25 W |
| United States | Boston | English | 42 22 N | 071 02 W |
| United States | Chicago | English | 41 59 N | 087 54 W |
| United States | Dallas | English | 32 54 N | 097 02 W |
| United States | Denver | English | 39 52 N | 104 40 W |
| United States | Detroit | English | 42 14 N | 083 20 W |
| United States | Honolulu | English | 21 21 N | 157 56 W |
| United States | Los Angeles | English | 33 56 N | 118 24 W |
| United States | Miami | English | 25 54 N | 080 17 W |
| United States | Minneapolis | English | 44 53 N | 093 13 W |
| United States | New York City | English | 40 46 N | 073 59 W |
| United States | Philadelphia | English | 39 53 N | 075 15 W |
| United States | Portland | English | 45 36 N | 122 36 W |
| United States | San Francisco | English | 37 37 N | 122 23 W |
| United States | Seattle | English | 47 27 N | 122 18 W |
| United States | St Louis | English | 38 42 N | 090 41 W |
| United States | Washington D.C. | English | 38 57 N | 077 27 W |
