# Supplementary material for: Do Seasons Have an Influence on the Incidence of Depression? The Use of an Internet Search Engine Query Data as a Proxy of Human Affect
Source: PLoS One. 2010 Oct 28;5(10):e13728. doi: 10.1371/journal.pone.0013728 (PMC2965678; doi:10.1371/journal.pone.0013728)
Supplement: Table S2 — List of equivalent words representing the search term “depression” in non-English languages. (0.05 MB DOC) [file pone.0013728.s002.doc]

**Table S2**. List of equivalent words representing the search term “depression” in non-English languages

| **Keyword** | **Language*** |
| --- | --- |
| 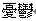 | Chinese** |
| depression | Deutsch |
| masennus | Finish |
| dépression | French |
| depresi | Indonesian |
| 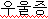 | Korean |
| depresjon | Norwegian |
| depresja | Polish |
| depressão | Portuguese |
| depresión | Spanish |
| depression | Swedish |

* In alphabetical order of English name of the corresponding language

** Traditional Chinese characters were used in Taiwan
