# Supplementary material for: Do Seasons Have an Influence on the Incidence of Depression? The Use of an Internet Search Engine Query Data as a Proxy of Human Affect
Source: PLoS One. 2010 Oct 28;5(10):e13728. doi: 10.1371/journal.pone.0013728 (PMC2965678; doi:10.1371/journal.pone.0013728)
Supplement: Table S3 — Cross-correlation coefficient between seasonal IMF of local search trend and that of temperature as well as solar radiation. (0.01 MB PDF) [file pone.0013728.s003.pdf]

Table S3. Cross-correlation coefficient between seasonal IMF of local search trend and that of temperature as well as solar radiation

Country	Representative Area/City	Latitude	Cross-correlation coefficient between local search trend and below variables	
			Temperature	Solar radiation
Argentina	Buenos Aires	34 35 S	-0.617	-0.811
Australia	New South Wales	33 52 S	-0.763	-0.784
Australia	Queensland	27 23 S	-0.730	-0.785
Australia	South Australia	34 56 S	-0.669	-0.692
Australia	Victoria	37 49 S	-0.835	-0.832
Brazil	Bahia	12 54 S	-0.556	-0.631
Brazil	Minas Gerais	19 48 S	-0.574	-0.693
Brazil	Parana	25 31 S	-0.748	-0.656
Brazil	Rio de Janeiro	22 55 S	-0.697	-0.846
Brazil	Rio Grande do Sul	30 00 S	-0.667	-0.700
Brazil	Santa Catarina	27 40 S	-0.766	-0.754
Brazil	Sao Paulo	23 37 S	-0.604	-0.822
Canada	Alberta	51 06 N	-0.806	-0.859
Canada	British Columbia	49 18 N	-0.874	-0.908

Canada	Ontario	43 37 N	-0.944	-0.902
Canada	Quebec	45 28 N	-0.801	-0.800
Chile	Puerto Montt	41 25 S	-0.844	-0.825
Colombia	Bogotá	04 42 N	-0.286	-0.384
Costa Rica	San Jose	10 00 N	-0.261	-0.207
Ecuador	Quito	00 09 S	-0.083	-0.355
Finland	Helsinki	60 15 N	-0.724	-0.744
France	Entire region	46 00 N	-0.582	-0.635
Germany	Entire region	52 28 N	-0.819	-0.899
Indonesia	Jakarta	06 06 S	-0.333	-0.529
India	Entire region	28 35 N	-0.616	-0.623
South Korea	Seoul	37 34 N	-0.639	-0.646
Mexico	Mexico City	19 24 N	-0.322	-0.517
Norway	Oslo	60 12 N	-0.769	-0.721
Portugal	Lisbon	38 43 N	-0.780	-0.587
Poland	Warsaw	52 10 N	-0.795	-0.824
Peru	Lima	12 00 S	-0.645	-0.506
Switzerland	Entire region	47 00 N	-0.511	-0.600
Spain	Entire region	40 00 N	-0.844	-0.861
Sweden	Stockholm	62 00 N	-0.868	-0.918

Taiwan	Entire region	25 02 N	-0.270	-0.366
United Kingdom	England	51 30 N	-0.600	-0.748
United Kingdom	Scotland	55 57 N	-0.645	-0.840
United States	Atlanta	33 39 N	-0.762	-0.816
United States	Boston	42 22 N	-0.818	-0.871
United States	Chicago	41 59 N	-0.760	-0.758
United States	Dallas	32 54 N	-0.658	-0.599
United States	Denver	39 52 N	-0.685	-0.747
United States	Detroit	42 14 N	-0.655	-0.742
United States	Honolulu	21 21 N	-0.344	-0.322
United States	Los Angeles	33 56 N	-0.477	-0.604
United States	Miami	25 54 N	-0.551	-0.630
United States	Minneapolis	44 53 N	-0.742	-0.762
United States	New York City	40 46 N	-0.836	-0.889
United States	Philadelphia	39 53 N	-0.773	-0.831
United States	Portland	45 36 N	-0.649	-0.750
United States	San Francisco	37 37 N	-0.706	-0.739
United States	Seattle	47 27 N	-0.725	-0.790
United States	St Louis	38 42 N	-0.535	-0.645
United States	Washington D.C.	38 57 N	-0.782	-0.872
