# Supplementary figures and images for: Do Seasons Have an Influence on the Incidence of Depression? The Use of an Internet Search Engine Query Data as a Proxy of Human Affect
Source: PLoS One. 2010 Oct 28;5(10):e13728. doi: 10.1371/journal.pone.0013728 (PMC2965678; doi:10.1371/journal.pone.0013728)

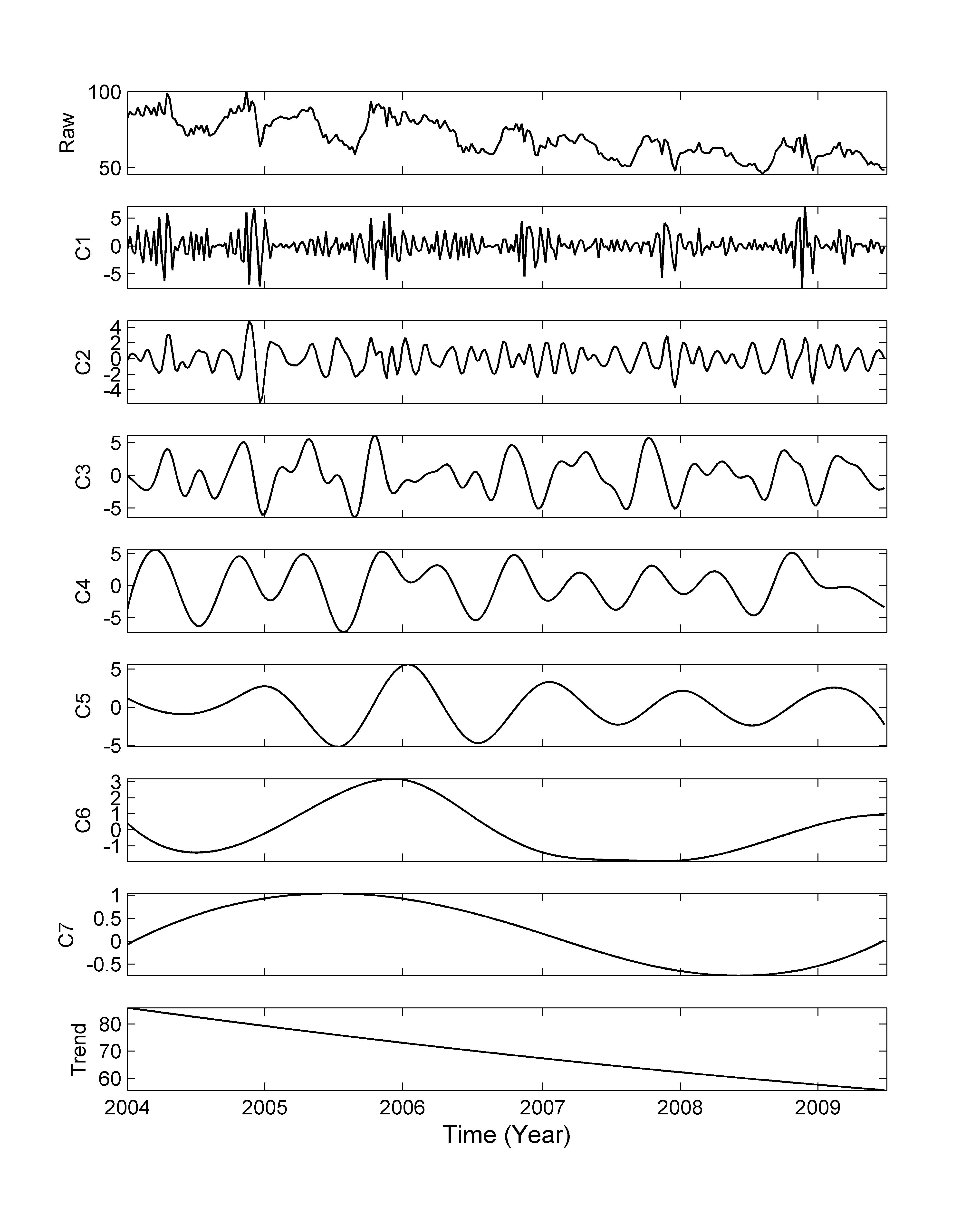

Supplement: Figure S1 — Empirical mode decomposition of search interest time series for depression within the United States, Jan 1 2004 through Jun 30 2009. (2.27 MB TIF) [file pone.0013728.s004.tif]
