# Supplementary material for: Do Seasons Have an Influence on the Incidence of Depression? The Use of an Internet Search Engine Query Data as a Proxy of Human Affect
Source: PLoS One. 2010 Oct 28;5(10):e13728. doi: 10.1371/journal.pone.0013728 (PMC2965678; doi:10.1371/journal.pone.0013728)
Supplement: Dataset S1 — Raw dataset of search trends of depression. (0.04 MB ZIP) [file pone.0013728.s006.zip › Supplementary dataset/Supplementary dataset.doc]

**Supplementary dataset**

**Do seasons have an influence on the incidence of depression? The use of an internet search engine query data as a proxy of human affect.**

Albert C. Yang^1,2,3^, Norden E. Huang^4^, Chung-Kang Peng^5^ and Shih-Jen Tsai^2,6^

^1^ Department of Psychiatry, Chu-Tung Veterans Hospital, Hsin-Chu County 31064, Taiwan

^2^ Divisions of Psychiatry, School of Medicine, National Yang-Ming University, Taipei 11217, Taiwan

^3^ Institute of Clinical Medicine, National Yang-Ming University, Taipei 11217, Taiwan

^4^ Research Center for Adaptive Data Analysis, National Central University, Chungli 32001, Taiwan

^5^ Division of Interdisciplinary Medicine and Biotechnology and Margret and H. A. Rey Institute for Nonlinear Dynamics in Medicine, Beth Israel Deaconess Medical Centre/Harvard Medical School, Boston, MA 02215 USA and Wyss Institute for Biologically Inspired Engineering at Harvard University, Boston, MA 02215, USA.

^6^ Department of Psychiatry, Taipei Veterans General Hospital, Taipei 11217, Taiwan

**Note:**

1. Supplementary files contain time series data of weekly search trends for depression (or equivalent words) from January 1, 2004 to June 30, 2009. Data were downloaded via Google Insights for Search ([www.google.com/insights/search/](http://www.google.com/insights/search/)) on Aug 18, 2009.
2. The most recent search trend data can be accessed via Google Insights for Search website.

Raw dataset of search trends of depression

| **Country** | **Representative Area/City** | **Filename** |
| --- | --- | --- |
| Argentina | Buenos Aires | AG_ALL_GIS.TXT |
| Australia | Entire region | AU_ALL_GIS.TXT |
| Australia | New South Wales | AU_NSW_GIS.TXT |
| Australia | Queensland | AU_QSL_GIS.TXT |
| Australia | South Australia | AU_SOA_GIS.TXT |
| Australia | Victoria | AU_VIC_GIS.TXT |
| Brazil | Bahia | BZ_BAH_GIS.TXT |
| Brazil | Minas Gerais | BZ_MIG_GIS.TXT |
| Brazil | Parana | BZ_PAR_GIS.TXT |
| Brazil | Rio de Janeiro | BZ_RDJ_GIS.TXT |
| Brazil | Rio Grande do Sul | BZ_RGS_GIS.TXT |
| Brazil | Santa Catarina | BZ_SAN_GIS.TXT |
| Brazil | Sao Paulo | BZ_SAP_GIS.TXT |
| Canada | Alberta | CA_ALB_GIS.TXT |
| Canada | British Columbia | CA_BCL_GIS.TXT |
| Canada | Ontario | CA_ONT_GIS.TXT |
| Canada | Quebec | CA_QBC_GIS.TXT |
| Chile | Puerto Montt | CH_ALL_GIS.TXT |
| Colombia | Bogotá | CL_ALL_GIS.TXT |
| Costa Rica | San Jose | CR_ALL_GIS.TXT |
| Ecuador | Quito | EQ_ALL_GIS.TXT |
| Finland | Helsinki | FI_ALL_GIS.TXT |
| France | Entire region | FR_ALL_GIS.TXT |
| Germany | Entire region | GM_ALL_GIS.TXT |
| Indonesia | Jakarta | ID_ALL_GIS.TXT |
| India | Entire region | IN_ALL_GIS.TXT |
| South Korea | Seoul | KO_ALL_GIS.TXT |
| Mexico | Mexico City | MX_ALL_GIS.TXT |
| Norway | Oslo | NW_ALL_GIS.TXT |
| Portugal | Lisbon | PG_ALL_GIS.TXT |
| Poland | Warsaw | PL_ALL_GIS.TXT |
| Peru | Lima | PR_ALL_GIS.TXT |
| Switzerland | Entire region | SL_ALL_GIS.TXT |
| Spain | Entire region | SP_ALL_GIS.TXT |
| Sweden | Stockholm | SW_ALL_GIS.TXT |
| Taiwan | Entire region | TW_ALL_GIS.TXT |
| United Kingdom | England | UK_ENG_GIS.TXT |
| United Kingdom | Scotland | UK_SCT_GIS.TXT |
| United States | Entire region | US_ALL_GIS.TXT |
| United States | Atlanta | US_ATL_GIS.TXT |
| United States | Boston | US_BOS_GIS.TXT |
| United States | Chicago | US_CGO_GIS.TXT |
| United States | Dallas | US_DAL_GIS.TXT |
| United States | Denver | US_DEN_GIS.TXT |
| United States | Detroit | US_DET_GIS.TXT |
| United States | Honolulu | US_HWI_GIS.TXT |
| United States | Los Angeles | US_LOS_GIS.TXT |
| United States | Miami | US_MIA_GIS.TXT |
| United States | Minneapolis | US_MIN_GIS.TXT |
| United States | New York City | US_NYC_GIS.TXT |
| United States | Philadelphia | US_PHL_GIS.TXT |
| United States | Portland | US_POR_GIS.TXT |
| United States | San Francisco | US_SAN_GIS.TXT |
| United States | Seattle | US_SEA_GIS.TXT |
| United States | St Louis | US_SLO_GIS.TXT |
| United States | Washington D.C. | US_WAS_GIS.TXT |
